# Subjective and Real Time: Coding Under Different Drug States

**Published:** 2015

**Authors:** Hugo Sanchez-Castillo, Kathleen M. Taylor, Ryan D. Ward, Diana B. Paz-Trejo, Maria Arroyo-Araujo, Oscar Galicia Castillo, Peter D. Balsam

**Affiliations:** Universidad Nacional Autónoma de México, México; Columbia University, U.S.A; University of Otago, New Zealand; Universidad Nacional Autónoma de México, México; Universidad Nacional Autónoma de México, México; Universidad Iberoamericana, México; Columbia University, U.S.A

**Keywords:** Timing, Temporal Information Processing, Acquisition, Recall, Haloperidol, Methamphetamine, dopamine

## Abstract

Organisms are constantly extracting information from the temporal structure of the environment, which allows them to select appropriate actions and predict impending changes. Several lines of research have suggested that interval timing is modulated by the dopaminergic system. It has been proposed that higher levels of dopamine cause an internal clock to speed up, whereas less dopamine causes a deceleration of the clock. In most experiments the subjects are first trained to perform a timing task while drug free. Consequently, most of what is known about the influence of dopaminergic modulation of timing is on well-established timing performance. In the current study the impact of altered DA on the acquisition of temporal control was the focal question. Thirty male Sprague-Dawley rats were distributed randomly into three different groups (haloperidol, d-amphetamine or vehicle). Each animal received an injection 15 min prior to the start of every session from the beginning of interval training. The subjects were trained in a Fixed Interval (FI) 16s schedule followed by training on a peak procedure in which 64s non-reinforced peak trials were intermixed with FI trials. In a final test session all subjects were given vehicle injections and 10 consecutive non-reinforced peak trials to see if training under drug conditions altered the encoding of time. The current study suggests that administration of drugs that modulate dopamine do not alter the encoding temporal durations but do acutely affect the initiation of responding.

Organisms are constantly extracting information from the temporal structure of the environment, which allows them to select appropriate actions and predict impending changes (Body et al., 2009; Body, Kheramin, Ho, Miranda Herrera, Bradshaw, & Szabadi, 2004; Buhusi & Meck, 2005). The mechanism underlying the perception, encoding, and retrieval of temporal regularities in the seconds and minutes range is referred to as interval timing (Boisvert & Sherry, 2006; Balsam, Sanchez-Castillo, Taylor, Van Volkinburg & Ward, 2009; Chiang, Al-Ruwaitea, Ho, Bradshaw & Szabadi, 1998).

One influential proposal about how temporal information guides behavior is Scalar Timing Theory (STT) (Gibbon, Church, & Meck, 1984). There are three general stages in this model: a clock stage, a memory stage, and a decision stage. The model hypothesizes that the clock stage is composed of a pacemaker and accumulator. When a stimulus is presented, the accumulation of pacemaker pulses is assumed to form the perceptual basis of subjective time. This representation of time is transferred to memory at the end of a stimulus. On future occasions when that stimulus is encountered a representation of its duration can be retrieved and compared to the current elapsing time to guide when a particular action will be appropriate (e.g., press a lever, stop responding or switch responses) (for a review see Buhusi & Meck, 2002 or Balsam et al., 2009). For example, if an animal is always rewarded for responding 16 s after the beginning of a trial, the expected time of reward (16 s after trial onset) will be stored in memory. On subsequent trials the elapsed time can be compared to the remembered time of reward. When that comparison crosses a threshold criterion for proximity to the expected time of reward, the animal will begin to make the previously rewarded response. Similarly, if the animal is not rewarded at 16 s on some trials, the elapsed time will exceed the remembered time and the animal will stop responding presumably due to the crossing of the same threshold. Thus the comparison of elapsed time to a remembered time of reward will govern the animal’s decision about when to start responding and when to stop trying to earn a reward on each trial. One other important aspect of STT is that the variability of estimates increases with the duration of the interval being timed. Specifically, this theory makes a precise prediction referred to as the scalar property that the standard deviation of temporal estimates increases linearly with the mean (Allman, Teki, Griffiths, & Meck, 2014; Gibbon et al., 1984).

Several lines of research have suggested that interval timing is strongly modulated by the dopaminergic system (Agostino, Golombek, & Meck, 2011; Balci, 2014; Meck, 1996). One explanation of this modulation is that dopamine modulates clock speed. The administration of dopaminergic agonists, such as amphetamine, causes behavior to occur earlier as shown in a leftward shift of the timing functions (Maricq, Roberts, & Church, 1981; Matell, Bateson, & Meck, 2006) consistent with a speeding of the internal clock, whereas the administration of dopaminergic antagonists, such as haloperidol, produces a rightward shift of the timing functions, consistent with what one would expect if the clock speed were slowed (MacDonald & Meck, 2005; Meck, 1983, 1986). It has also been suggested that since dopamine affects motivation that the modulation of timed behavior might involve changes in the thresholds to start behavior rather than timing, per se (Balci, Ludvig, Abner, Zhuang, Poon, & Brunner, 2010; Balci, Ludvig, & Brunner, 2010; Balci, 2014; Taylor, Horvitz, & Balsam, 2007). In this motivational view, changes in the initiation of behavior might contribute to the shifts in average timing functions.

In most experiments on timing, the subjects are first trained to perform a timing task while drug free. Consequently, most of what is known about the influence of dopaminergic modulation of timing is on well-established timing performance. In contrast, the current experiment manipulated DA signaling pharmacologically during the acquisition phase of a timing task. This is a critical manipulation if DA does affect clock speed. If subjects are trained with DA agonists they will learn the time with a speeded clock but this will not be manifest in performance: If they encode time with a fast clock, the values stored in memory will consistently map to real time as decoded by a fast clock. Similarly, subjects who are given DA antagonists might encode time with a slow clock but because they are measuring elapsed time with the same clock speed (i.e., slow clock) their performance is expected to be accurate in real time. Interestingly, if subjects were then tested without drugs (with normalized clock speed), those subjects who were trained with a fast clock would respond later in real time (it would take longer to reach a time encoded by a fast clock) while those trained with the antagonist would be expected to respond earlier (it would take less time to reach a time encoded by a slow clock).

The current experiment evaluated the impact of altering DA signaling on temporal encoding by training subjects after injections of either d-amphetamine or haloperidol from the very beginning of training on interval timing tasks. The subjects were first trained on a fixed interval (FI) task in which they were rewarded for the first response after a fixed amount of time elapsed from the onset of a trial followed by training on a Peak Interval (PI) timing task. In the PI task subjects continued to be rewarded on FI trials but these FI trials are intermixed with peak trials (3 or 4 times longer) in which no reward is presented. When data are averaged over peak trials subjects show a bell-shaped pattern of responses, in which the maximum response rate (the peak time) typically occurs around the time at which the reinforcement was delivered. In addition to the peak time, the PI procedure yields measures of response vigor (peak rate), timing variability (peak spread). Additionally, the trial-by-trial performance of individual subjects can be analyzed to examine any differential timing effects of drugs on the initiation (start time) or termination (stop time) of responding (Balci, 2014; Taylor et al., 2007). A change in timing will be reflected in comparable shifts in start and stop times. For example, if a clock runs 10% faster then both start and stop times should occur 10% earlier. In contrast, if motivation to engage in the task is altered, this might affect the likelihood of response initiation reflected in start times without necessarily affecting the stop times. In the latter case, d-amphetamine and haloperidol might decrease and increase start times, respectively, but have no effect or even the opposite effect on stop times if they alter the motivation to engage in the timing task (Balci, 2014). Furthermore, there would be no reason to expect acute motivational effects to be changed by extended training.

Another possible effect of speeding or slowing an internal clock is in the effect that this might have on timing variability. A major source of variability in temporal processing is thought to occur during storage of the number of accumulated pulses into long term memory (Gibbon et al., 1984). The level of variability is thought to be proportional to the number of pulses that are stored. Consequently times encoded by a fast clock (more accumulated pulses) and then stored in long term memory will have greater variability than times encoded by a slow clock (fewer accumulated pulses). If amphetamine speeds an internal clock then times encoded under amphetamine might be more variable than times encoded under vehicle and times encoded with a haloperidol induced slowing of the clock might be less variable than those encoded in the non-drug state.

Subjects were trained on the FI and PI tasks under a drug or vehicle followed by a drug free test of timing in which no reward was given. The training phase of the experiment permits the assessment of both transient and stable effects of drug administration and the drug free test phase allows for the assessment of whether or not clock speed was altered by drugs during the initial training as described above. If haloperidol slows clock speed and amphetamine increases clock speed during initial training, when tested drug free with clocks running at normal speed, the haloperidol trained subjects should respond earlier and the amphetamine trained subjects should respond later than subjects trained on vehicle (normal clock speed).

## Method

### Subjects

Thirty experimentally naïve 12-week old male CD rats (Crl:CD (SD), Sprague-Dawley Derivate, Charles Rivers, Wimington, MA.) were obtained and housed in polypropylene home-cages (two per cage) under a 12:12 dark:light cycle. At the beginning of the experiment, all subjects had access to food and water ad libitum. The subjects were weighed and handled for one week to habituate them. Once the weight baseline was established (272.7 SD 38.5 gr), the subjects were food-restricted until they reached a stable 85% of their initial weight. All animal research protocols were approved by Institutional Animal Care and Use Committees (IACUC) of Columbia University and the New York State Psychiatric Institute.

### Drugs

D-amphetamine sulfate (SIGMA, St. Louis, MO) was dissolved in an isotonic saline solution and administrated intraperitoneally (i.p.) at 1.0 mg/kg dose. Haloperidol sulfate (SIGMA, St. Louis, MO) was dissolved in an isotonic saline with 0.5 % lactic acid solution and then administrated i.p. at 0.12 mg/kg dose. All doses were selected because of their efficacy in previous timing studies (see the introduction) and were administered 15 min before the experimental session in a volume of 1 ml/kg.

### Apparatus

Ten operant chambers were used in this study (Model ENV-008, Med Associates, Inc. St. Albans, VT). Each box was 30.5×24.1×21.0 cm with a grid floor of 19 stainless steel rods. A food magazine (3.0×4.0 cm) was situated 2 cm above the floor in the center of the right wall. An automatic food dispenser was used to deliver 45-mg food pellet reinforcers (Bio-serv, Frenchtown, NJ). Each box had two retractable levers located 4 cm above the floor and 2 cm from both sides of the food magazine. Each box was situated in a sound-attenuating chamber (75.0×61.0×38.0 cm) with a ventilating fan. Stimulus presentation and data recording were controlled by a PC with MedPC-IV software (Med Associates, Inc. St. Albans, VT).

### Procedure

#### Feeder training

The rats were initially feeder-trained for three sessions (20 min). During those sessions, a variable-time 45 s reinforcement schedule dispensed pellets at irregular intervals. The experimental sessions were conducted 5 days per week at approximately the identical time each day.

#### Lever press training

When the subjects finished feeder training, lever press training began. During the first two sessions one of the levers (location counterbalanced across subjects) was inserted into the chamber. A food pellet was delivered 1 s after the insertion of the lever. The lever remained extended into the chamber for 30 s, and any presses were reinforced by additional pellets. The lever was then retracted and a new ITI was initiated. A variable inter-trial interval (ITI) with a mean duration of 125 s separated the trials. The ITI durations were approximately exponentially distributed with a truncated range (2 s-240 s). The sessions were programmed to run for 60 min or until the subject had earned 100 reinforcers. On the next two days, no free pellets were delivered and the subjects were allowed to obtain up to 100 pellets in 60 min on a fixed ratio 1 (FR1) schedule. No injections occurred during the initial feeder training and lever press training.

#### FI training

When all rats acquired the lever pressing behavior, they were randomly distributed into four groups: saline (*n* = 5), saline mixed with lactic acid (*n* = 5), d-amphetamine (*n* = 10) or haloperidol (*n* = 10). Each animal received an i.p. injection 15 min before the beginning of the experimental session. For the next five consecutive sessions all groups were trained on a FI 16 s schedule timed from the onset of a trial, which was signaled by a tone of 4500 Hz (80 dB). Trials were separated by the same variable ITI with a mean of 125 s used during lever press training.

#### Peak interval training

Peak interval (PI) trials were introduced following the FI training phase. During this phase, as in the FI training, each animal received a daily i.p. injection 15 min before the start of the experimental session. The pharmacological conditions for each subject remained identical to the FI training. Each peak trial was signaled by the same cue as the FI trial but it lasted for 64 s (4 times the FI) and no rewards could be earned. The number of PI trials was increased over sessions. During PI sessions 1 to 11, the proportion of FI to PI trials was 80/20. During PI sessions 12 to 16, the proportion was 60/40, and finally, during PI sessions 17 to 24, the proportion was 50/50. PI sessions 1–21 were used to examine the acquisition of temporal control in all groups and sessions 22–24 were used as the final baseline against which test performance was compared.

#### Test session

In the test session no drugs were administered, instead of d-amphetamine or haloperidol subjects were injected with their respective vehicles, and tested for 10 consecutive PI trials. No rewards were presented in the test session.

### Data Analysis

Each individual’s responses over time was collected in 1-sec bins and fit using the Sigma Plot 11 software with a three-parameter Gaussian function (f=a*exp(.5*((x−x0)/b)^2)), where a is the maximum response; b is the standard deviation (SD) and x0 is the peak time. The Weber Fraction was obtained, by dividing the standard deviation by the peak time. Relative response rates were computed by dividing rates by the maximum response rate for each subject. For the trial-by-trial analysis, the start and stop time on each trial was obtained by using the algorithm described in Taylor et al. (2007). The midpoint of the interval between the start and the stop is referred to as the middle time and used as the subject’s estimate of the expected time of reinforcement on that trial. The difference between start and stop times is referred to as the spread.

There was no difference between vehicle groups in any phase of the experiment. Thus, the data of the two control groups were pooled for subsequent analyses. The results obtained from the vehicle, haloperidol and d-amphetamine groups during the training and test phases were evaluated with mixed model ANOVAs in which all within-subject comparisons were treated as repeated measures. All significant results were followed with Tukey’s post-hoc comparisons.

## Results

### Peak Acquisition

**Figure 1:**
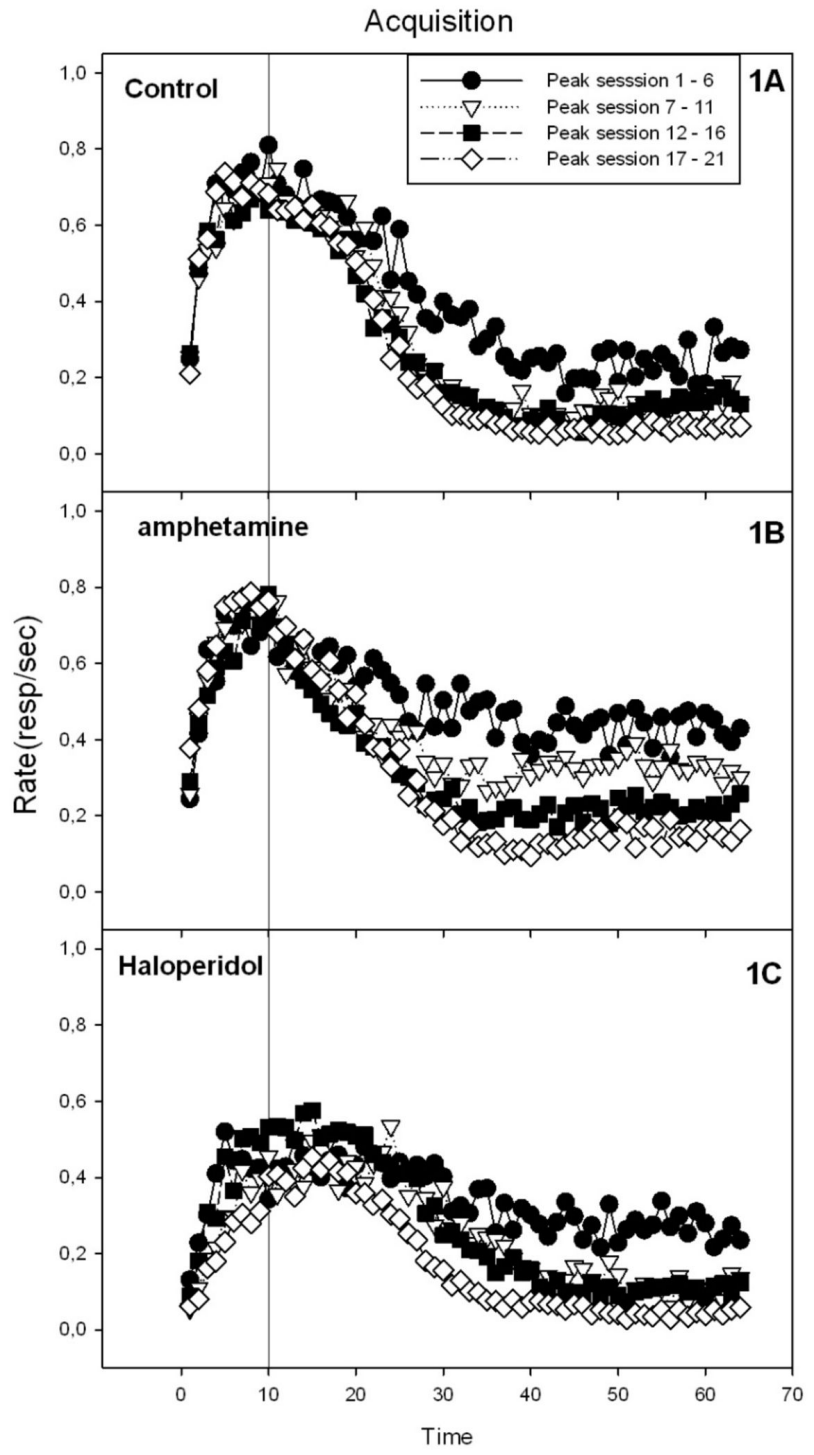
Acquisition under the d-amphetamine, haloperidol or vehicle treatment on the PI task. 1A) Control group acquisition, 1B) d-amphetamine group acquisition, 1C) haloperidol group acquisition during training sessions.

**Figure 2:**
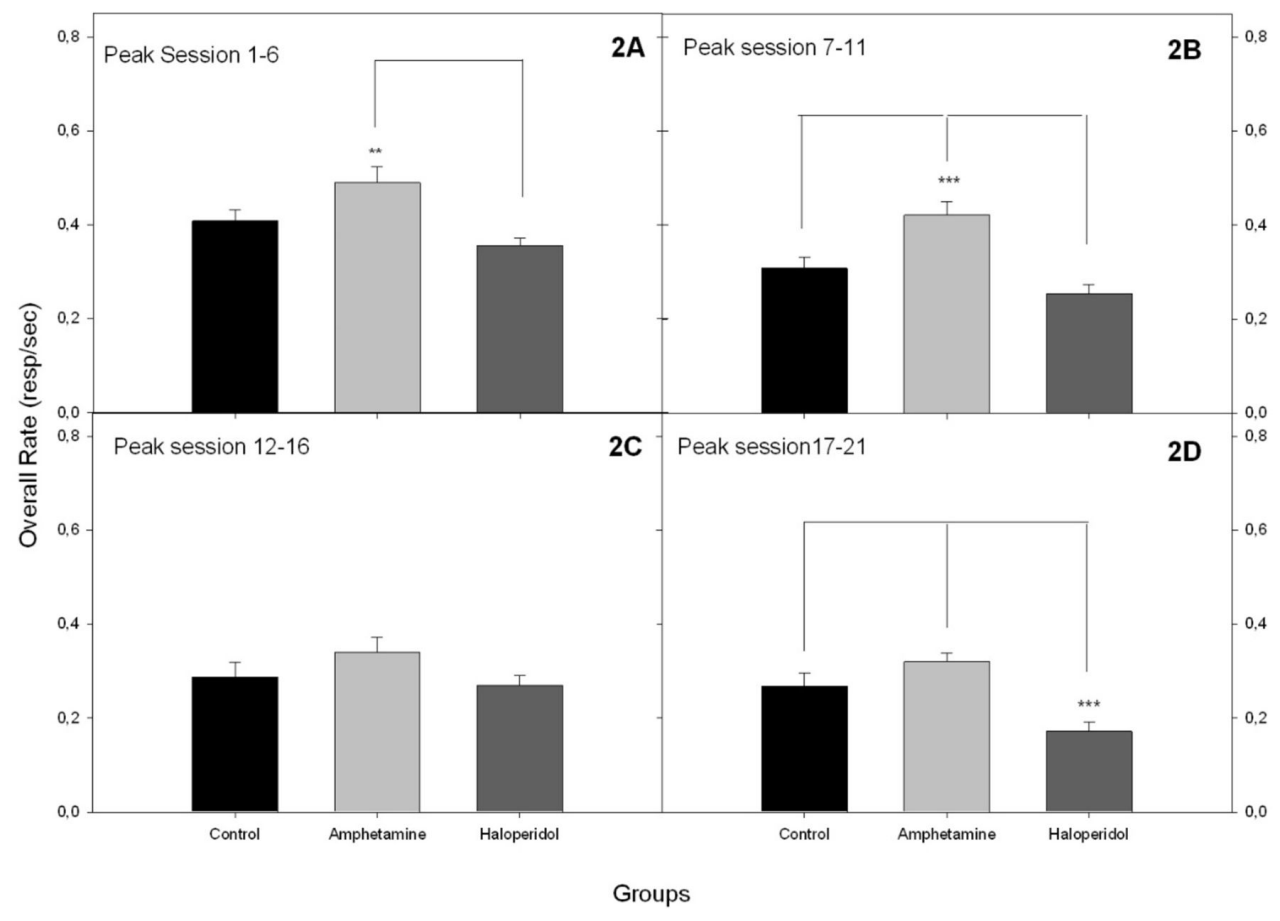
The overall rate during the development of the acquisition in the peak procedure task in blocks of six sessions. 2A) A significant increase of the overall rate in the d-amphetamine group is observed in the first block (1–6), but only in comparison to the haloperidol group. 2B) The overall rate of the d-amphetamine group was higher than the control or haloperidol groups in the second block (7–11). 3C) The overall rate effect showed no differences between the three groups in the third block (12–16). 3D) The haloperidol group showed a lower response rate compared to the control and d-amphetamine groups in the fourth block (17–21).

The acquisition of performance on the PI procedure task is shown in Figure 1. All groups showed temporal control during the first block of training. However, only about half the subjects had good Gaussian fits in the first block. By the end of training all but three subjects’ peak trials were well described by the Gaussian fits. Only those subjects for which the fit yielded a peak time greater than zero were included in the comparison of the Gaussian parameters across groups. In a Group x Block ANOVA on peak rates there was a significant effect of group, *F*(2, 14) = 8.39, *p* < 0.01, as well as a nearly significant Group x Block interaction, *F*(6, 42) = 2.31, *p* = 0.051. In every block, there was a significant difference between groups in peak rates. Post hoc tests showed that the vehicle and haloperidol group differed in every block, while the d-amphetamine and haloperidol groups differed in peak rate during blocks 2 and 4. The vehicle and d-amphetamine groups did not differ statistically in any of the blocks (see Table 1). Response rate differences during training were examined in more detail by computing the overall response rates during the entire PI trial. Figure 2 shows the mean overall response rate for each drug condition in each of the training blocks. Overall rates decreased significantly over blocks, *F*(3, 81) = 64.13, *p* < 0.01, and differed significantly between groups, *F*(2, 27) = 9.05, *p* < 0.01, and showed a significant Group x Block interaction, *F*(6, 81) = 2.71, *p* < 0.01. Follow-up ANOVAs in each block showed a significant difference between groups in the first block arising from significantly higher rates in the d-amphetamine group compared to the haloperidol group. A similar pattern was evident in the second block where the post hoc test showed that the d-amphetamine group responded at a higher rate than both other groups. By the third block the groups were no longer significantly different in overall response rate (though the averages showed a similar trend to the previous block). In the fourth block the groups again differed significantly but now the overall group difference arose from lower response rates in the haloperidol group compared to the vehicle and amphetamine groups, which did not differ.

The analysis of the other parameters of the Gaussian fits showed that the SD declined significantly over the course of training in all groups, *F*(3, 39) = 13.62, *p* < 0.01. There was also a significant difference between groups, *F*(2, 13) = 4.44, *p* < 0.05. The post-hoc tests indicated that there was a significantly lower SD in the vehicle group compared to the d-amphetamine group. With respect to peak time, there was no systematic change over blocks but there was Group x Block interaction, *F*(6, 39) = 2.85, *p* < 0.01. From the second block on, the haloperidol group had later peak times than the other two groups, which did not differ.

**Figure 3:**
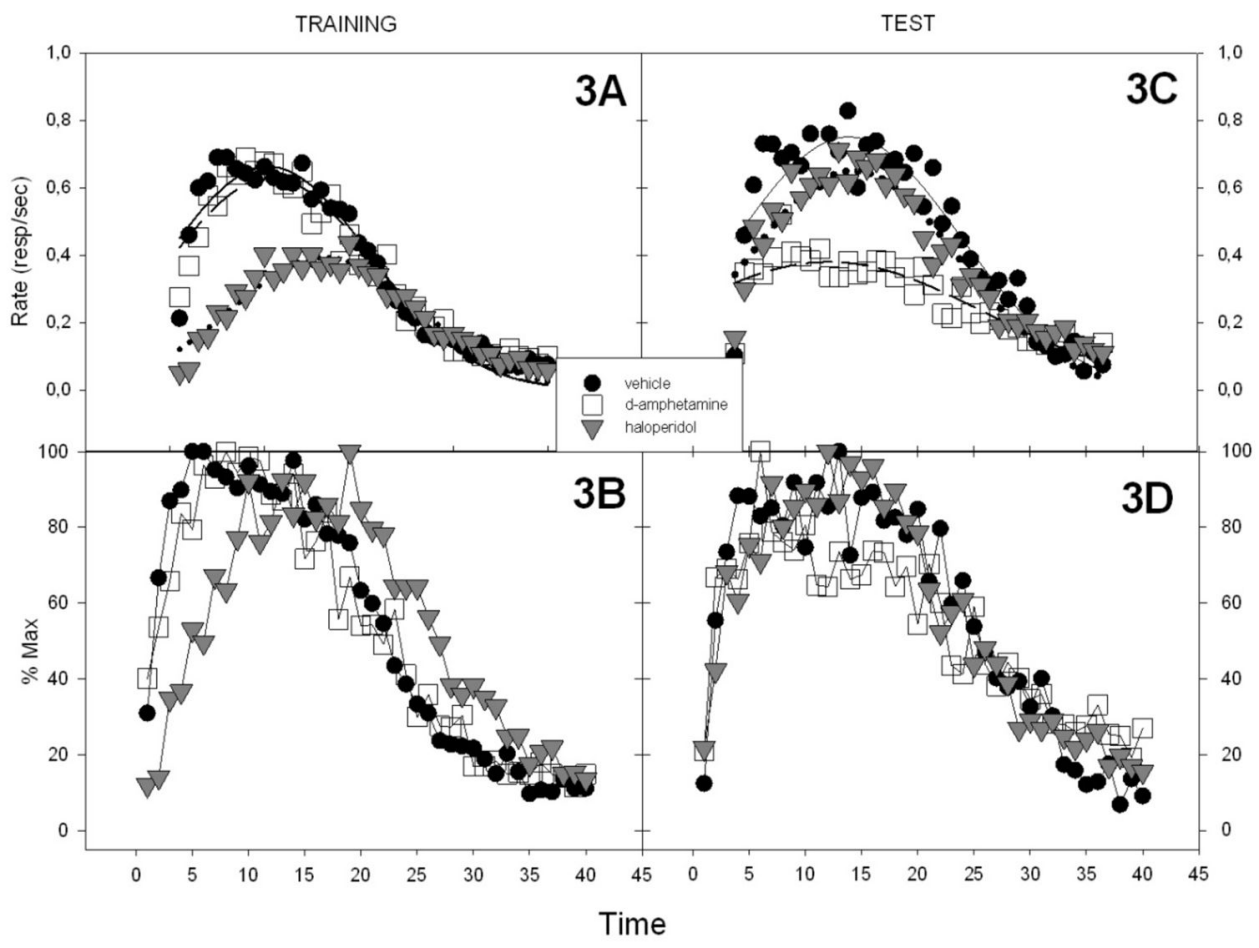
The training, test fittings and relative rates for all groups. All the fits were performed with a three-parameter Gaussian equation (f=a*exp(−.5*((x−x0)/b)^2)). 3A) Gaussian fits to the training data for all groups. 3B) The relative response rates (% of the maximum response) in the training conditions. 3C) The Gaussian fits to the test data for all groups. 3D) The relative response rates for the the test sessions (% of the maximum response).

**Figure 4:**
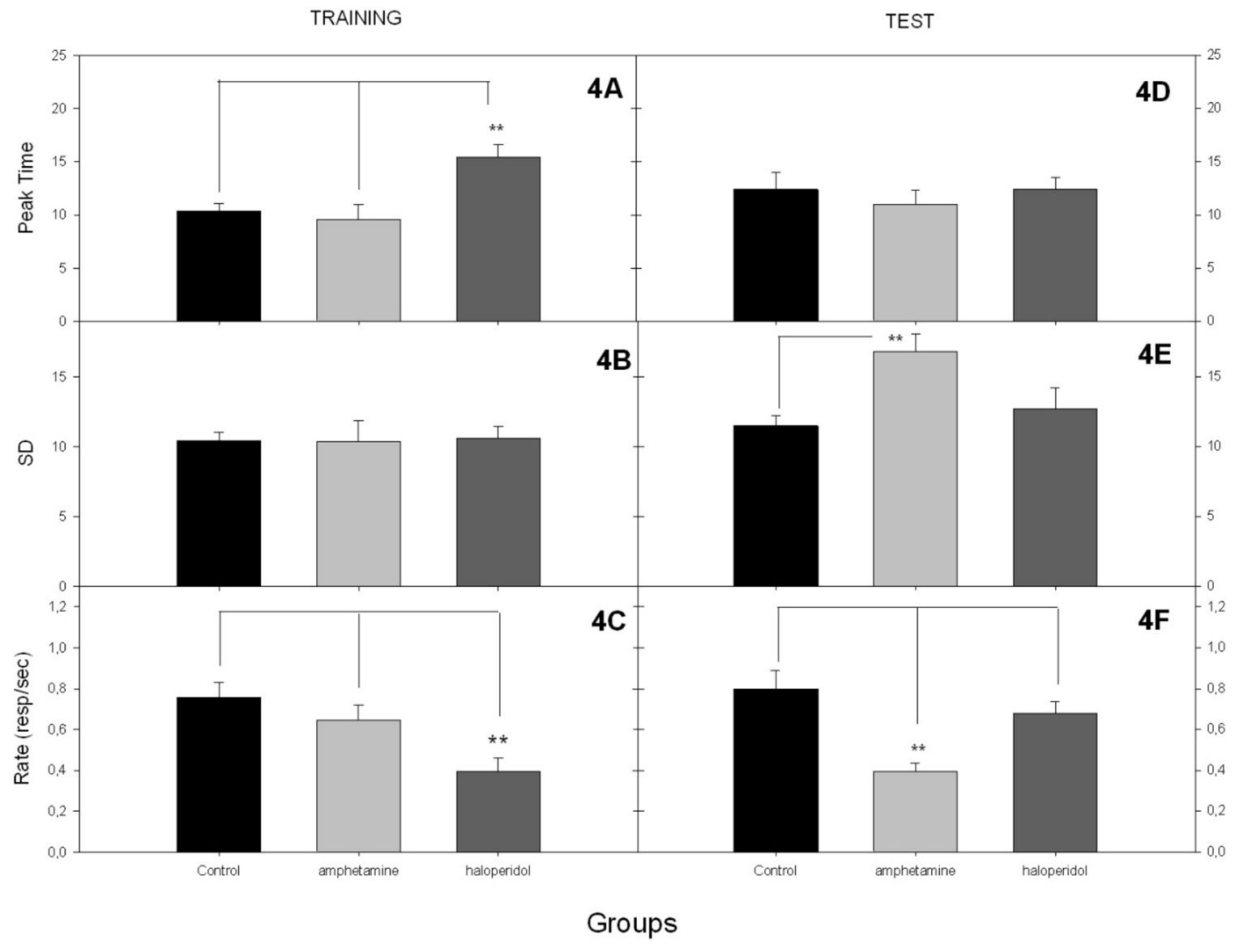
The timing measures obtained from the three-parameter Gaussian Equation (f=a*exp(−.5*((x−x0)/b)^2)). 4A) The peak times (x0) during the training phase. 4B) The standard deviation (b) during the training phase. 4C) The response rates (a) during the training phase. 4D) The peak times during the test phase. 4E) The standard deviation during the test phase. 4F) The response rate during the test.

Figure 3 shows the PI trial performance during the last three days of training and the single test session in which all subjects received vehicle. This plot also shows that haloperidol lowered response rates and shifted responding to later times during training. This rightward shift produced by haloperidol is evident when plotted as relative response rates (3B). During the test (3C, D) all groups have roughly comparable peak times but surprisingly the d-amphetamine group responded at a lower rate than the other two groups. Figure 4 shows the parameters for the Gaussian fits. With respect to the peak rates, a two-way ANOVA with phase (training vs testing) and group as factors showed a significant effect of phase, *F*(1, 27) = 5.53, *p* < 0.01, group, *F*(2, 27) = 7.37, *p* < 0.01, as well as a Group x Phase interaction, *F*(2, 27) = 15.43, *p* < 0.01. Follow-up ANOVAs showed that the groups differed significantly in both phases. At the end of the training phase there were significantly lower peak rates in the haloperidol group than the other two groups. During the drug-free test phase the difference between groups arose from the d-amphetamine group having significantly lower rates than the other two groups.

With respect to standard deviations the two-way ANOVA showed no main effects but there was a significant Group x Phase interaction, *F*(1, 21) = 3.59, *p* < 0.05. During training there was no difference between groups in SD, *F*(2, 23) = 1.00, *p* > 0.05; however, during the test there was a significant difference between groups, *F*(2, 25) = 4.70, *p* < 0.05, because the d-amphetamine group had greater SDs than the other groups but this was only significant in the comparison with the vehicle group.

A two-way ANOVA on Peak Times yielded a significant effect of group, *F*(2, 21) = 4.92, *p* < 0.05, and a significant Group x Phase interaction, *F*(2, 21) = 4.51, *p* < 0.05. During the training phase, peak times differed across groups, *F*(2, 23) = 14.20, *p* < 0.01. The haloperidol group had significantly later peak times compared to the other groups, which did not differ. During the drug-free test phase, peak time did not differ significantly between groups, *F*(2, 25) < 1.00, *p =* 0.67.

**Figure 5:**
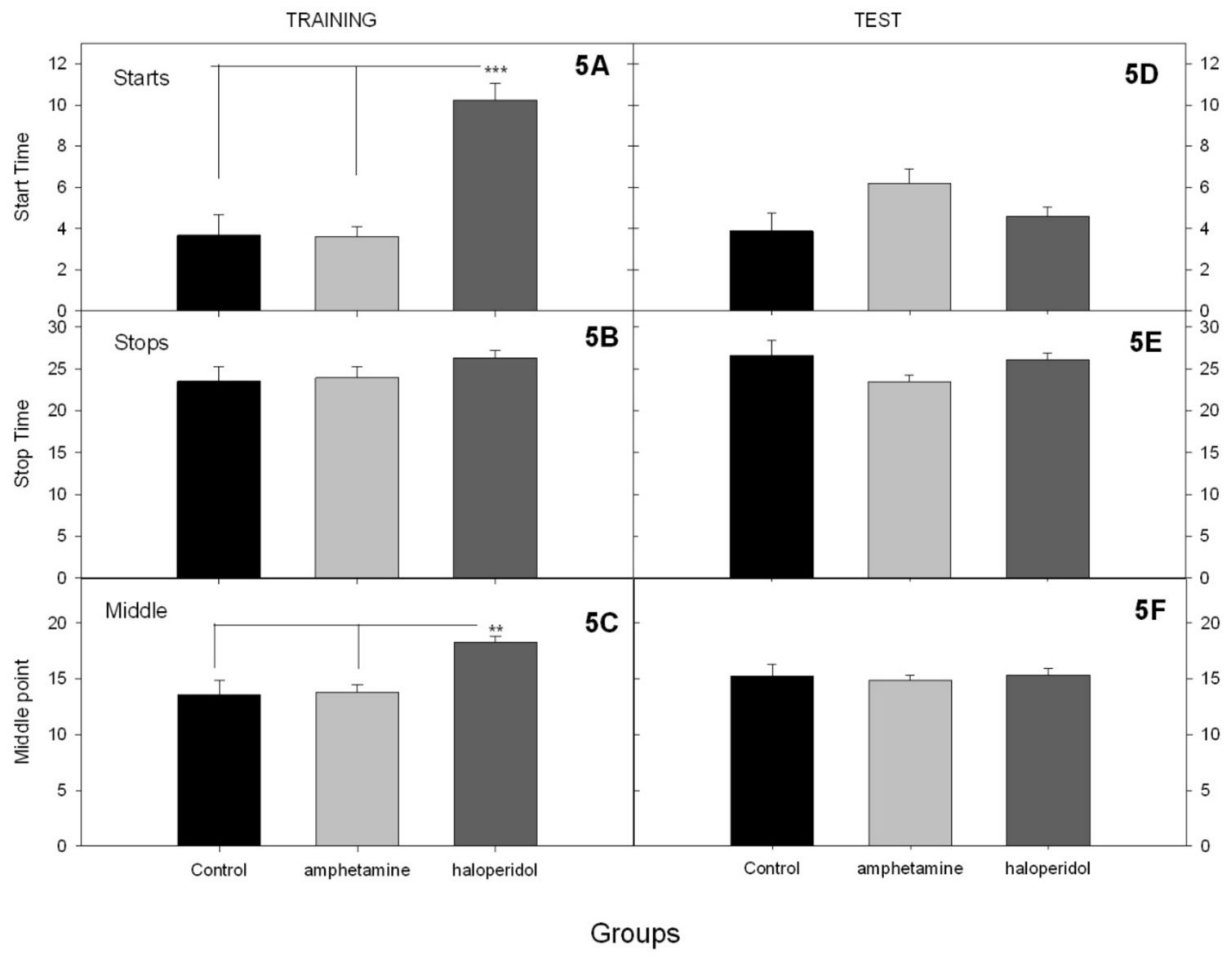
The start and stop times for all conditions during the training and test sessions. Start times in the training (5A) and the test (5D) phase. Stop times during the training (5B) and test (5E) phase. Middle times in the training (5C) and test (5F) phase. ** asterisk marks a *p* < 0.05, whereas *** marks a *p* < 0.001.

Because average peak curves do not always reflects the trial-by-trial performance, the points at which responding started and stopped on every trial were identified. Figure 5 shows the start and stop analyses at the end of peak training and during the test. A two-way ANOVA on start times with group and phase as factors yielded a significant effect of group, *F*(2, 27) = 33.70, *p* < 0.0001, significant effect of phase, *F*(1, 27) = 5.19, *p* < 0.05, and a significant Group x Phase interaction, *F*(2, 27) = 33.70, *p* < 0.0001. Simple main effects analyses showed that at the end of training, the groups differed significantly in start time, F(2, 27) = 25.395, *p* < 0.0001, because the haloperidol group had significantly later start times than the vehicle and d-amphetamine groups (5A). During the vehicle test session the groups also differed in start time, *F*(2, 27) = 4.05, *p* < 0.05, but now it was because the vehicle and amphetamine groups differed. The interaction was followed up by testing whether start times changed across phases in any of the groups. Start times did not differ across phases in the vehicle group. However, start times were significantly later under the vehicle test than during d-amphetamine training, *T*(9) = 4.32, *p* < 0.005, and significantly earlier during vehicle test than during haloperidol training, *T*(9) = 5.25, *p* < 0.001). The two-way ANOVA on stop times did not detect any significant differences between groups as a function of group or phase.

The impact of the drug treatments on the variability of start and stop times was also examined. During training there was a significant difference between groups in the SD of start times, *F*(2, 27) = 10.81, *p* < 0.001, as well as in stop times, *F*(2, 27) = 7.16, *p* < 0.005. The haloperidol group was significantly more variable in both start and stop times than the other two groups, which did not differ. There was also a significant difference in variability between groups during the test phase in both start, *F*(2, 2) = 5.29, *p* < 0.05, and stop times, *F*(2, 27) = 13.00, *p* < 0.001. During the test phase, the d-amphetamine treated subjects were more variable than the vehicle group in start times and more variable than both the vehicle and haloperidol groups in stop times. The vehicle and haloperidol groups did not differ from one another in either start or stop time variability during testing. In timing tasks variability of time estimates increases with the mean of the interval being estimated. Consequently, in order to compare variability in start and stop times across durations, coefficients of variation were computed for each subject by dividing each SD by the subject’s mean duration for the corresponding start and stop times. The relative variability of start times was far greater than stop times in every group, *F*(1, 27) = 92.13, *p* < 0.001. The mean CV for start times was 0.91 and it was 0.32 for stop times across all conditions. During training the groups did not differ in CV of start times but the CV of stop times was significantly different across groups, *F*(1, 27) = 3.73, *p* < 0.05. The stop time CV’s in the haloperidol group were significantly greater than those of the vehicle group during training. During testing, the CV of start times did not differ across groups but the CV of stop times did differ, *F*(1, 27) = 19.13, *p* < 0.001, as a result of a greater stop time CV in the d-amphetamine group than in both the vehicle and haloperidol groups, which did not differ.

## Discussion

The purpose of the current study was to examine the effects of altering dopamine function on the acquisition and performance of timed behavior. The administration of d-amphetamine had little impact on timing during training except to generate broader timing distributions early in training. Additionally, there was no difference between the d-amphetamine group and vehicle group in start or stop times during the training phase. When tested drug free, the Gaussian parameters of timing of the d-amphetamine group were no different than the Gaussian parameters of subjects exposed to vehicle throughout training. However, the single trials analysis indicated that start times were significantly later in d-amphetamine group compared to the vehicle-trained subjects. Furthermore, when the d-amphetamine group was tested on vehicle their start times significantly increased compared to the earlier phase. Similarly, haloperidol appeared to have little effect on timing per se. During training the Gaussian fits indicated that haloperidol resulted in later peak times. However, when the data were analyzed on a trial-by-trial basis haloperidol also only affected the time at which responding started, not the time at which it stopped on PI trials. When this group was tested on vehicle their Gaussian parameters did not differ from the other groups but their start times shifted to a significantly earlier time.

These results were surprising in light of the expectation that drug effects on clock speed during training would be revealed during the drug-free testing. Previous work indicated that at least one effect of these drugs would be to alter the speed of the internal clock. Several reports have shown timing displacements to the left after the administration of dopaminergic agonists (Bizot, 1997; Body et al., 2006; Buhusi & Meck, 2002; MacDonald & Meck, 2005; Maricq & Church, 1983; Maricq et al., 1981; Matell et al., 2006), indicative of increased clock speed, and displacements to the right, indicative of slowed clock speed, after the administration of dopaminergic antagonists (Buhusi & Meck, 2002; Drew, Fairhurst, Malapani, Horvitz, & Balsam, 2003; MacDonald & Meck, 2005; Maricq & Church, 1983). Consequently, subjects given d-amphetamine would be expected to encode time with a fast clock and subjects given haloperidol would be expected to encode time with a slowed clock. During training, since each subject would use the same clock for encoding and decoding time, any clock speed effects would not be manifest in performance. However, during the drug free test, with clocks running at normal speed, the amphetamine trained subjects should respond at later times and haloperidol trained subjects respond at earlier times than the control subjects. When tested drug free, only start times shifted and these shifts were consistent with the predictions of the clock speed hypothesis (later start times in the d-amphetamine group and earlier start times in the haloperidol group). However, stop times did not change across phases. It is not clear why if clock speed had been altered by the drugs that stop times would not be as affected as start times. Thus, the most parsimonious explanation of the effect of both d-amphetamine and haloperidol is that they acutely affected the initiation of responding rather than a timing process.

The failure to find a clock speed effect might have occurred for a number of reasons. One possibility is that long-term exposure to the drugs permanently altered clock speed. Perhaps, whatever impact the drugs had on clock speed during the training phase permanently altered the timing process. Consequently, when tested drug free, the clock with altered speed, did not readjust to the original non-drug state. Since the clock would be identical during training and testing, no clock speed effect on peak times would be detected. Another possibility is that when subjects are given extensive training, their timing becomes less susceptible to DA modulation (Horvitz, Choi, Morvan, Eyny, & Balsam, 2007; Mead, 1974). Finally, it is also possible that DA modulation does not affect clock speed, per se, but rather alters the tendency to initiate actions. Often, the data obtained from the PI procedure is analyzed by fitting a Gaussian curve to the average performance across trials. The best fits though can be strongly influenced by when subjects begin or end their responding on individual trials (Balci, 2014; Taylor et al., 2007). In our training data, the Gaussian fits indicated that the haloperidol group had later peak times but the individual trials analysis showed that this was attributable to late starts and unchanged stops relative to the other groups. If haloperidol had altered the representation of the expected time of reward then we would expect subjects to both begin responding later and to stop responding later in this condition. Because only start times were affected by haloperidol, caution is called for in interpreting the Gaussian fits of peak response curves as purely reflecting underlying timing.

The low response rate in the haloperidol group during training is anticipated by a substantial literature showing that DA antagonists lower response rates on many operant schedules by lowering reward value (Wise, 2004, 2008) and/or by reducing the effort that animals are willing to expend to obtain reward (Salamone & Correa, 2012). On the other hand, the lowered response rates of the d-amphetamine group during testing was an unanticipated but interesting aspect of the current results. To the extent that tonic dopamine levels influence motivation (Salamone, Correa, Mingote, & Weber, 2003), the lowered rates may represent contrast of a condition of high DA (usually associated with high reward) and a low DA condition (usually associated with a downshift in reward). Alternatively, the lower rate may be the result of CRs evoked by the context associated with d-amphetamine (Shen, Meredith, & Napier, 2006), which compete with the operant response. Lastly, the change from training with d-amphetamine to drug-free testing might have increased the speed of extinction during the test session either as a direct result of withdrawal or because the contextual cues (ongoing drug effects) were different during training and testing. The increase in timing variability in the d-amphetamine group during testing could be the consequence of the rapid extinction or the shift in motivational state (Jensen, Stokes, Paterniti, & Balsam, 2013).

The single trials analysis further suggests that factors unrelated to temporal processing affect the start and stop times on individual trials. The scalar property as reflected in an approximately constant CV across a broad range of intervals is often observed in timing research (Allman, Teki, Griffiths, & Meck, 2014). The CV for start times was about three times larger than that of stop times suggesting that there are sources of variability in start times that do not affect stops. Perhaps, factors related to the initiation of action and/or termination of competing activities uniquely contribute to start variability. During training haloperidol did not change the CV of start times but increased the CV of stop times suggesting a selective impact of this drug on processes underlying the cessation of action. Similarly, during the drug free testing the d-amphetamine trained group showed a selective increase in the CV of stop times. To the extent that stop times are a purer reflection of timing variability than start times it is possible that the haloperidol directly affected timing variability during training while amphetamine withdrawal affected it during testing. Another speculation is that in both cases the increased variability in stop times could reflect trial-to trial variability in attention to time once the subject has started responding (Fortin, Bedard, & Champagne, 2005) but we have no direct evidence that speaks to either explanation.

It is clear that there is still much to untangle in understanding DA modulation of timed behavior. Some papers report a clear leftward shift with dopaminergic agonists (Buhusi & Meck, 2002; Cevik, 2003; Cheng, MacDonald, & Meck,2006; Eckerman et al., 1987; Kraemer, Randall, Dose, & Brown, 1997; Meck & Church, 1983; Meck, 1983) and a rightward shift after antagonist administration (Buhusi & Meck, 2002; Drew et al., 2003; MacDonald & Meck, 2005; Meck, 1986). Other studies have observed the opposite effects with agonists (Bayley, Bentley, & Dawson, 1998; Santi, Coppa, & Ross, 2001) or no effect on timing with antagonists (Cheung et al., 2006). Moreover, there are additional studies indicating that the administration of dopaminergic drugs can have inconsistent effects on clock speed (Chiang et al., 2000; McClure, Saulsgiver, & Wynne, 2011; Cheung et al., 2006) as well as effects on response initiation (Balci, 2014; Balci et al., 2010a, b; Taylor et al., 2007) and attention (Buhusi & Meck, 2002). The wide range of outcomes likely arises from the use of different interval timing tasks (Saulsgiver, McClure, & Wynne, 2007, Chiang et al., 2000; Body et al., 2013), training histories (Saulsgiver, McClure, & Wynne, 2006, Odum, 2002; Orduna, Garcia, & Bouzas, 2012) and species (Odum & Ward, 2007; Chiang et al., 2000; Drew et al., 2007; Abner, Edwards, Douglas, & Brunner, 2001) as well as from differences in specific pharmacological methods. The current study indicates that administration of drugs that modulate dopamine from the start of training appear to affect response initiation so long as the drugs are present. The boundary conditions for the DA modulation of timing need to be defined.

## Figures and Tables

**Table 1:** Standard Deviation (SD), rate and peak times obtained after the fit with the 3 parameter Gaussian function

Treatment	Measure	Blocks							
		1		2		3		4	

		Mean	SD	Mean	SD	Mean	SD	Mean	SD
Vehicle	Rate	6.02	0.32	0.83	0.30	0.84	0.23	0.88	0.36
	SD	24.42	9.00	11.16	3.16	11.46	3.63	12.88	7.45
	Peak Time	7.97	10.51	13.75	2.16	11.98	3.15	11.27	3.01
d-amph	Rate	0.68	0.30	0.90	0.52	0.76	0.29	1.05	0.34
	SD	55.26	39.17	40.63	27.12	23.22	17.66	10.85	5.06
	Peak Time	5.04	52.96	9.60	5.34	10.40	2.19	10.55	3.22
Hal	Rate	0.44*	0.09	0.43*	0.15	0.51*	0.19	0.38*	0.17
	SD	38.71	15.57	16.62	3.88	15.92	5.32	11.75	4.86
	Peak Time	14.01	12.76	19.62	3.09	15.56	3.06	16.52	1.55

* marks significant differences against the vehicle condition (*p* < 0.05)
